# Diagnostic and prognostic utility of mid-expiratory flow rate in older community-dwelling persons with respiratory symptoms, but without chronic obstructive pulmonary disease

**DOI:** 10.1186/s12890-015-0081-4

**Published:** 2015-07-31

**Authors:** Gülmisal Güder, Susanne Brenner, Stefan Störk, Matthias Held, Berna DL Broekhuizen, Jan-Willem J Lammers, Arno W Hoes, Frans H Rutten

**Affiliations:** Julius Center for Health Sciences and Primary Care, University Medical Center Utrecht, Utrecht, The Netherlands; Department of Internal Medicine I, Cardiology, University Hospital Würzburg, Oberdürrbacherstr. 6, D-97080 Würzburg, Germany; Comprehensive Heart Failure Center, University of Würzburg, Würzburg, Germany; Medical Mission Hospital, Department of Internal Medicine, Respiratory Medicine and Cardiology, Würzburg, Germany; Department of Respiratory Medicine, University Medical Center Utrecht, Utrecht, The Netherlands

## Abstract

**Background:**

The maximal expiratory flow at 50 % of the forced vital capacity (MEF_50_) is the flow where half of forced vital capacity (FVC) remains to be exhaled. A reduced MEF_50_ has been suggested as a surrogate marker of small airways disease. The diagnostic and prognostic utility of this easy to assess spirometric variable in persons with respiratory symptoms, but without COPD is unclear.

**Methods:**

We used data from the UHFO-COPD cohort in which 405 community-dwelling persons aged 65 years or over, and a general practitioner’s diagnosis of chronic obstructive pulmonary disease (COPD) underwent pulmonary function testing and echocardiography. In total 161 patients had no COPD according to the spirometric GOLD criteria. We considered MEF_50_ as reduced if < 60 % of predicted.

**Results:**

Of the 161 patients without COPD (mean age 72 ± 5.7 years; 35 % male; follow-up 4.5 ± 1.1 years), 61 (37.9 %) had a reduced MEF_50_. They were older, had more pack-years of smoking, more respiratory symptoms, and used more frequently inhaled medication than the remaining 100 subjects. A reduced MEF_50_ was nearly twice as often associated with newly detected heart failure (HF) at assessment (29.5 % vs. 15.6 %, p = 0.045). In age-and sex-adjusted Cox regression analysis, a reduced MEF_50_ was significantly associated with episodes of acute bronchitis (hazard ratio 2.54 95 % confidence interval (1.26; 5.13) P = 0.009), and in trend with pneumonia (2.14 (0.98; 4.69) P = 0.06) and hospitalizations for pulmonary reasons (2.28 (0.93; 5.62) P = 0.07).

**Conclusions:**

In older community-dwelling persons with pulmonary symptoms but without COPD, a reduced MEF_50_ may help to uncover unrecognized HF, and identify those at a higher risk for episodes of acute bronchitis, pneumonia and hospitalizations for pulmonary reasons. Echocardiography and close follow-up should be considered in these patients.

## Background

The maximal expiratory flow at 50 % of the forced vital capacity (MEF_50_) is the flow where half of forced vital capacity (FVC) remains to be exhaled [1]. It corresponds to the forced expiratory flow at 50 % (FEF_50_) and correlates highly with the maximum mid-expiratory flow (FEF25-75 %) [2]. As such, MEF_50_ indicates obstruction of small airways and may be used as a surrogate of early small airways disease defined by an abnormally low mid-expiratory flow in the presence of normal forced expiratory volume in 1 second (FEV_1_), FVC, and FEV_1_/FVC ratio [3].

The usefulness of forced expiratory flows to diagnose small airways disease is, however controversial, as intra-individual variability is high, even in healthy subjects [4, 5]. Especially in the presence of central obstructive ventilatory disorders such as COPD or asthma, forced expiratory flow parameters lack reproducibility and do not correlate well with other variables related to airway obstruction (as forced vital capacity, FVC and residual volume-total lung capacity index, (RV/TLC) [6, 7]. However, in absence of central obstructive disorders, e.g. in patients with symptoms suggestive of COPD but in whom COPD has been excluded spirometrically, a low maximal expiratory flow and small airways disease may strongly suggest an alternative diagnosis, e.g. primary bronchiolar disorders (bronchiolitis) or interstitial lung diseases with bronchiolar involvement [8]. Moreover, a low maximum expiratory flow might also direct differential diagnosis towards a non-pulmonary condition causing respiratory symptoms such as heart failure, either known or latent [9].

The aim of our analysis was to assess the prevalence and the potential prognostic value of a low post-dilatory MEF_50,_ in older community-dwelling individuals with pulmonary symptoms of airways obstruction, but without COPD.

We assessed the relation of a low post-dilatory MEF_50_ with hitherto unknown heart failure, first episode of either acute bronchitis, pneumonia, hospitalizations for pulmonary reason, or all-cause mortality.

## Methods

### Study design and population

We made use of a data set derived from a prospective cohort study (UHFO-HF) investigating 405 patients aged ≥65 years with a clinical diagnosis of COPD in primary care. Population and study characteristics have been described in detail previously [10]. Patients clinically suspect of COPD but without spirometrically verified obstruction were recruited from 51 primary care practices and assessed in the outpatient clinic of the University Medical Center Utrecht, Netherlands. A standardized clinical examination, extensive pulmonary function testing (PFT), chest radiography, and echocardiography were performed in all participants [10]. Exclusion criteria comprised an already established diagnosis of heart failure, severe psychiatric disorder, immobility, and terminal illness with short life expectancy precluding study participation [10].

In 244 of 405 (60 %) patients from the original cohort COPD defined as a post-bronchodilator ratio of FEV1/FVC <0.7 according to the GOLD criteria could be spirometrically verified [11].

The present analysis refers to the remaining 161 patients with exclusion of a COPD diagnosis in spirometry. The study complied with the Declaration of Helsinki, and the Medical Ethics Committee of the University Medical Center Utrecht approved the study protocol. All participants gave their written informed consent.

### Missing values

In four patients post-bronchodilator MEF_50_ values were missing. In these patients pre-bronchodilator measurements were used (two subjects with a MEF_50_ < 60 %, and two with a MEF_50_ > 60 % of predicted). Thus, the present study analyzes 161 patients without COPD.

### Pulmonary function tests and definition of low MEF_50_

Pre-and post-bronchodilator pulmonary function testing was performed with a fixed-volume bodyplethysmograph (Masterlab Jaeger, Würzburg, Germany). The post-bronchodilator test was assessed 30 minutes after inhalation of 40 μg of ipratropium bromide.

There are no published guidelines regarding normal values for MEF_50_. We used 60 % of post-dilatory MEF_50_ predicted as cut-off in the presence of a post-dilatory FEV1/FVC ≥ 0.7 for defining low MEF_50_ [6]. Following the recommendations of the European Respiratory Society we used age, gender, and height for the calculation of the predicted values of all lung function parameters [12].

### Blood test results

Laboratory results such as white blood cell count, haemoglobin, haematocrit, and serum aminoterminal pro-hormone B-type natriuretic peptide (NT-proBNP) were measured as part of the study assessment.

### Echocardiography and definition of heart failure

Echocardiography was performed by two experienced cardiac sonographers using a Philips Sonos 5500 imaging system (Andover, MA, USA) and interpreted by a single cardiologist, who was blinded to clinical data [10]. Left ventricular ejection fraction (LVEF) was derived using Simpson’s biplane or, when not possible, by single plane area–length method [13].

Left atrial volume was calculated by the biplane area–length method from apical four-and two-chamber views and indexed to the body surface area [14]. Mitral inflow and pulmonary venous inflow were measured with pulsed-wave Doppler echocardiography. Tissue Doppler was used to assess the sub-mitral movement of the septal wall, and a composite of abnormalities in diastolic function was employed to define diastolic dysfunction [15].

Presence or absence of heart failure was established by an expert panel consisting of two cardiologists, a pulmonologist, and a general practitioner using all available diagnostic information, including echocardiography and pulmonary function tests [10]. Heart failure was classified by the panel in line with the recommendations of the ESC guidelines on heart failure. The definition required presence of signs and symptoms suggestive of heart failure and additional criteria that led to the following subtypes: a) “heart failure with a reduced ejection fraction” when LVEF was reduced (arbitrarily defined as ≤45 %); b) “heart failure with preserved ejection fraction” when echocardiographically determined diastolic dysfunction was present and LVEF >45 %; of note, symptoms and/or signs should not, or insufficiently, be explained by co-incident pulmonary disease; and c) “right-sided heart failure” (cor pulmonale) when LVEF was >45 %, and the calculated pulmonary artery pressure >40 mmHg [10].

### Follow-up and outcomes ascertainment

Patients included in the study were followed from April 2001 to June 2007 during a mean period of 4.3 (standard deviation 1.1) years. The follow-up data collection mode was described before [16]. In brief, the general practitioner’s (GP) electronic medical files were scrutinized to obtain information on patient’s drug prescription, pulmonary hospitalizations and survival status. Acute bronchitis was defined as an episode with bronchial wheezing and rhonchi for which the GP prescribed antibiotics and/or pulse prednisolon for 7 to 10 days.

Most episodes of pneumonia were diagnosed clinically by the GP and only occasionally confirmed by chest X-ray or sputum culture.

### Data analysis

Continuous data were expressed as mean (standard deviation, SD) or median (quartiles). Group comparisons between patients with and without a MEF_50_ < 60 % of predicted were performed using Fisher’s exact test or Mann–Whitney *U*-test, as appropriate (Tables 1 and 2). Correlations between MEF_50_ with other pulmonary function parameters were calculated with the Spearman rank order correlation coefficient (r). The strength of the correlation was graded using the following guide for the absolute value of r: 0.00-0.19 very weak; 0.20-0.39 weak; 0.40-0.59 moderate; 0.60-0.79 strong; 0.80-1.0 very strong.

**Table 1:** Characteristics of 161 patients with a GP’s diagnosis of COPD, but with a post-dilatorory FEV1/FVC ratio > 0.7, divided in those with MEF_50_ < vs ≥ 60 % of predicted

	All, N = 161	MEF_50_ < 60 % of predicted, N = 63	MEF_50_ ≥ 60 % of predicted, N = 98	*p*-value
Demographics				
Male sex, %	34.8	30.2	37.6	0.40
Age, years	72 (67; 76)	73 (69; 79)	71 (66; 74)	0.002
Pack years smoking, years	2.3 (0; 28)	10 (0; 38)	0.6 (0; 20)	0.026
Body mass index, kg/m^2^	27.0 (24.7; 30.0)	26.9 (24.7; 30.7)	27.1 (24.7; 29.7)	0.87
LVEF in %	60 (58; 60)	60 (56; 62)	60 (59; 60)	0.88
Systolic blood pressure, mmHg	154 (142; 164)	154 (144; 164)	153 (142; 165)	0.60
Comorbidities				
Hypertension; %	53.4	46.0	46.9	1.0
Atrial fibrillation, %	5.0	3.2	6.1	0.48
Diabetes, %	9.9	6.3	12.2	0.29
A history of asthma, %	12.4	14.3	11.2	0.63
Heart failure, %	20.5	28.6	15.3	0.048
Obesity	24.8	28.6	22.4	0.45
Symptoms				
Dyspnoea uphill, %	93.2	95.2	91.8	0.53
Dyspnoea while walking on level ground, %	43.5	58.7	33.7	0.002
Coughing >3 months, %	27.3	36.5	21.4	0.046
Wheezing, %	59	71.4	51.0	0.014
Phlegm production, %	42.9	49.2	38.8	0.20
Laboratory findings				
Hemoglobin, mmol/l	8.9 (8.3; 9.4)	8.9 (8.3; 9.2)	9.0 (8.4; 9.6)	0.24
Leucocytes /nl	7.3 (6.1; 8.7)	7.6 (6.8; 9.5)	7.0 (5.9; 8.2)	0.004
C-reactive protein, mg/l	3 (3; 6)	3.0 (3.0; 7.0)	3.0 (3.0; 5.8)	0.24
NT-proBNP, mg/dl	14.3 (8.1; 25.5)	16.8 (10.1; 29.3)	13.9 (7.0; 24.9)	0.078
Pharmacotherapy				
Diuretics	23.6	28.6	20.4	0.2
ACEi or ARB, %	23.6	25.4	22.4	0.71
Beta-blocker, %	13.7	11.1	15.3	0.49
Aspirin, %	23.0	27.0	20.4	0.34
Inhaled beta-2agonist, %	41.6	49.2	36.7	0.14
Inhaled anticholinergic, %	36	47.6	28.6	0.018
Inhaled corticosteroid, %	58.4	61.9	56.1	0.30
Chronic oral corticosteroid, %	1.9	4.8	0	0.058

Values are shown as median (quartiles) or percentages

Abbreviations: ACEi, angiotensin converting enzyme inhibitor; ARB, angiotensin II type 1 receptor blocker; obesity was defined as a body mass index ≥ 30 kg/m^2^

**Table 2:** Pulmonary function test results in patients with a MEF_50_ < vs ≥ 60 % of predicted

	All, N = 161	MEF_50_ < 60 % of predicted, N = 63	MEF_50_ ≥ 60 % of predicted, N = 98	*P* value
Post-dilator FEV1/FVC, %	76 (74; 81)	74 (71; 76)	80 (76; 85)	<0.001
FEV1, % of predicted	103 (89; 115)	85 (75; 102)	110 (101; 121)	<0.001
FVC, % of predicted	105 (91; 120)	93 (80; 112)	112 (100; 125)	<0.001
Post-dilator increase of FEV1 > 200 ml, %	13.7	17.5	11.2	0.35
Post-dilator increase of FEV1 > 200 ml and >12 %, %	11.8	14.3	10.2	0.46
Rtot, % of predicted	124 (86; 178)	165 (121; 213)	110 (76; 143)	<0.001
TLC, % of predicted	105 (97; 114)	103 (93; 112)	107 (97; 116)	0.18
TLC < 80 % of predicted, %	3.1	3.1	3.3	0.99
RV, % of predicted	108 (96; 126)	112 (100; 141)	107 (93; 120)	0.025
RV/TLC, %	102 (93; 115)	114 (101; 132)	98 (90; 106)	<0.001
DLCO, % of predicted	78 (67; 89)	70 (59; 80)	83 (75; 93)	<0.001
MEF_25_, % of predicted	54 (38; 76)	38 (30; 46)	68 (54; 93)	<0.001
MEF_50_, % of predicted	68 (49; 86)	47 (39; 55)	79 (70; 98)	<0.001
MEF_75_, % of predicted	94 (74; 111)	75 (58; 91)	104 (91; 122)	<0.001
PEF, % of predicted	104 (88; 121)	98 (73; 108)	111 (96; 129)	<0.001

Data are shown as median (quartiles) or percentages. All pulmonary function test variables refer to post-dilatory values and are presented as percentage of predicted or as percentages

Abbreviations: FEV1, forced expiratory volume in 1 second; FVC, forced vital capacity; RV, residual volume; Rtot, total resistance; TLC, total lung capacity; DLCO, diffusion capacity of carbon monoxide; MEF maximum expiratory flow 25/50/75 % of the FVC remain to be exhaled; PEF peak expiratory flow

Univariate, and age and sex adjusted multivariable Cox regression models were calculated for different outcomes (acute bronchitis, pulmonary hospitalizations, pneumonia, and all-cause mortality) and reported as hazard ratios (HR) with 95 % confidence intervals (CI). P values <0.05 were considered statistically significant. Data analysis was performed with SPSS 21 (IBM, Munich, Germany).

## Results

### Demographics of the patients with COPD according to the GOLD criteria

Patients excluded from our analysis (244 individuals with a GOLD diagnosis of COPD) were more often male (68 vs. 35 %, P < 0.001), and slightly older (73 [70; 77]) vs. 72 [67; 76] years P = 0.016) than the 161 patients included. All but one of the 243 patients with GOLD-COPD and available information on MEF had a post-dilatory MEF_50_ < 60 % of predicted confirming that a MEF_50_ below the chosen threshold is common in COPD.

### Demographics of the patients without COPD according to the GOLD criteria

Table 1 lists the baseline characteristics of the 161 individuals under study, stratified by MEF_50_ </≥ 60 % of predicted. Patients with a low MEF_50_ (38 %) were older, had more pack-years of smoking, suffered more often from respiratory complaints, used more often inhaled medication (especially anticholinergics), and had higher leucocyte counts than subjects with a MEF_50_ ≥ 60 %. Twenty-two of the 63 patients (35 %) with a low MEF_50_ < 60 % of predicted had a FEV1 and/or FVC % of predicted below 80 %. The remaining 41 participants (65 %) had normal values of FEV1 and FVC % of predicted.

### Correlation between MEF_50_ and other pulmonary function test results

Patients with a low MEF_50_ had a normal total lung capacity (TLC), while the carbon monoxide (CO) diffusion capacity was lower than in those with a MEF_50_ ≥ 60 % of predicted. Patients with a low MEF_50_ also had higher levels of total resistance, higher residual volume (RV) and larger RV/TLC ratio than subjects with a ‘normal’ MEF_50_ (Table 2). Reversibility of airway obstruction, defined as an increase of FEV1 levels > 200 ml and >12 % to baseline, did not differ between </≥ MEF_50_ 60 % of predicted.

Table 3 presents Spearman rank-order correlation coefficients for the association between MEF_50_ and other continuous pulmonary function test parameters. MEF_50_ correlated moderately to strongly with all pulmonary function test results, except for TLC (no correlation) and RV (very weak correlation).

**Table 3:** Correlation between MEF_50_ and other pulmonary function parameters

	FEV1/ FVC	FEV1	FVC	Rtot	TLC	RV	RV/TLC	DLCO	MEF25 %	MEF75 %	PEF
r	0.62^**^	0.64**	0.46**	-0.63**	0.13	−0.18*	−0.58**	0.50**	0.84**	0.76**	0.59**

Spearman’s correlation coefficients (r) between continuous values of MEF_50_ and other pulmonary function parameters (as % of predicted) with the Spearman’s rank correlation test

* refers to p < 0.05 and **to p < 0.001

### Association of low expiratory flow with newly detected heart failure

In total, N = 33 patients (20.5 %) were newly diagnosed with heart failure; N = 16 with heart failure with reduced ejection fraction, N = 17 with heart failure with preserved ejection fraction, and N = 0 isolated right-sided heart failure. A MEF_50_ < 60 % was associated with a new diagnosis of heart failure at the assessment: The incidence of newly diagnosed heart failure was 29.5 % in those with MEF_50_ < 60 % compared to 15.6 % in persons with a MEF_50_ ≥ 60 % of predicted (P = 0.045, Table 1).

Patients with a novel diagnosis of heart failure also had lower post-dilator MEF_75 %_ of predicted (84 % [64; 103 %] vs. 97 % [86; 119 %], P = 0.03) than the 128 patients without heart failure, whereas peak expiratory flow and post-dilator MEF_25 %_ were not different between these two groups. Patients with heart failure had more often FEV1 values below 80 % of predicted than subjects without heart failure (27 % vs. 12 %, P = 0.05). The corresponding FVC values were also numerically but not statistically different (FVC below 80 % of predicted 21 vs. 11 %, P = 0.15).

### Association of low MEF_50_ with prognosis

During follow-up, 34 patients experienced acute bronchitis and 27 patients a pneumonia, 21 patients were hospitalized for a pulmonary reason (pneumonia, bronchitis, acute exacerbation of newly diagnosed COPD), and 17 patients died (Table 4). In age-and sex-adjusted Cox regression analysis, a low MEF_50_ was significantly associated with acute bronchitis (2.54 (1.26; 5.13) P = 0.009) but not with pneumonia (2.14 (0.98; 4.69) P = 0.06), hospitalizations (2.28 (0.93; 5.62) P = 0.07, Table 5) or death (1.84 (0.68; 4.99) P = 0.23). If patients with heart failure (n = 33) were excluded from the analysis the associations became significant for all outcome measures except total mortality (Table 5).

**Table 4:** Number of pulmonary events and all-cause deaths during a mean follow up of 4.5 years in patients with and without MEF_50_ < 60 % of predicted

	All (N = 161)	MEF_50_ < 60 % N = 63	MEF_50_ ≥ 60 % N = 98	*P* value
Episodes of acute bronchitis, N (%)	34 (21.1 %)	20 (31.7 %)	14 (14.3 %)	0.01
Pneumonia, N (%)	27 (16.8 %)	15 (23.8 %)	12 (12.2 %)	0.08
Hospitalization for pulmonary reason, N (%)	21 (13.0 %)	12 (19.2 %)	9 (9.2 %)	0.09
All-cause death, N (%)	17 (10.6 %)	10 (15.9 %)	7 (7.1 %)	0.11

Absolute numbers of clinically relevant outcome measures during follow-up. *P* value refers to Fishers exact test

**Table 5:** Prognostic utility of MEF_50_ < 60 % of predicted in patients without COPD

	Acute bronchitis	Pneumonia	Hospitalization for pulmonary reasons	Death
Model 1	2.53 (1.28; 5.02) P = 0.008	2.11 (0.99; 4.51) P = 0.054	2.24 (0.94; 5.31) P = 0.07	2.17 (0.83; 5.71) P = 0.11
Model 2	2.54 (1.26; 5.13) P = 0.009	2.14 (0.98; 4.69) P = 0.06	2.28 (0.93; 5.62) P = 0.07	1.84 (0.68; 4.99) P = 0.23
Model 3	3.04 (1.36; 6.79) P = 0.007	2.66 (1.10; 6.40) P = 0.03	3.86 (1.25; 11.9) P = 0.019	1.97 (0.52; 7.36) P = 0.32

Hazard ratios with 95 % confidence intervals from Cox proportional hazard regression are presented. Median follow-up was 4.6 years. MEF_50_ < 60 % of predicted coded as “1”

Model 1: Unadjusted

Model 2: Adjusted for age and sex

Model 3: Adjusted for age and sex in the subgroup of 128 patients without heart failure

## Discussion

In this retrospective analysis of 161 older community-dwelling individuals with a clinical diagnosis of COPD but without spirometrically verified airways obstruction, we found that low maximum mid-expiratory flow (i.e., MEF_50_ < 60 % of predicted) was prevalent in 38 %. These patients had increased pulmonary resistance and residual volumes, and a higher RV/TLC index than subjects with a MEF_50_ ≥ 60 %. Further, a low MEF_50_ was associated with incident heart failure and predicted a higher risk for acute bronchitis during follow-up.

Small airways disease is frequently found in patients with airways obstruction as asthma or COPD [6]. In the present study we did not see differences in the prevalence of low MEF_50_ in patients with and without a history of asthma. However, since our patients were selected according to a GP’s diagnosis of COPD, the prevalence of patients with a history of asthma was low (12 % in the total cohort). Reduced MEF is a characteristic feature of COPD [4]: in the original cohort of patients with verified COPD (244/405, data not shown) according to the GOLD criteria, a MEF_50_ < 60 % of predicted was detected in 242 out of 243 persons (data not shown). In the remaining patient, MEF values were not available. The prognostic capacity of low MEF is thus not meaningful in patients with established airways obstruction.

Lower MEF values may also be found in the absence of airways obstruction, in primary bronchiolar disorders or interstitial lung disease, but also in systemic inflammatory diseases such as rheumatoid arthritis [8]. A low MEF is easily detectable with spirometry and relates to inflammation of the small non-cartilaginous bronchioles with an internal diameter of <2 mm and may well be an indicator of small airways disease or be considered as a precursor stage of COPD [6, 17, 18]. However, to determine the underlying pathophysiological substrate, histological investigation of lung biopsies or high resolution computer tomography scans would be needed, procedures that are not part of the routine assessment in clinical practice and thus were not available for this study [8, 19].

Expiratory flow volumes have previously been proposed as a tool for diagnosing extra-pulmonary restrictive disease [9]. Heart failure is such a condition as increased heart size and pulmonary congestion may simply reduce lung volumes [20]. In addition, pulmonary fluid overload in heart failure may cause or aggravate external bronchial obstruction with subsequent compromise of FEV1 [20, 21]. Thus, clinically and spirometrically, heart failure frequently mimics COPD. Indeed, in congestive heart failure, it has been repeatedly be shown that FEV1 values may be more reduced than corresponding FVC values, yielding FEV1/FVC ratios <0.70 in the absence of COPD [22, 23]. Importantly, this finding (i.e., pseudo-COPD) is not reproducible when the same individuals are re-assessed after recompensation under euvolemic conditions, calling for a more conscious use of spirometry in these cases [21, 24, 25]. Additionally, expiratory flow measurements may be more susceptible to heart failure than FEV1 or FEV1/FVC: in 29.5 % of the cases with a MEF_50_ < 60 % of predicted, previously unrecognized heart failure was detected. This is more than three times as high as might be expected in the general population aged > 65 years (estimated prevalence of heart failure in this age group 7-10 %) [26] and also higher than the percentage of newly detected cases of heart failure in the 244 patients with a GOLD-COPD diagnosis (prevalence 21 %) from the original cohort [10, 27]. Further 18 out of 33 patients (55 %) with previously unrecognized heart failure had a MEF_50_ < 60 % predicted, whereas only 9 (27 %) had a reduced FEV1 < 80 % predicted.

Although our study convincingly related a reduced MEF_50_ to clinically relevant outcomes, we acknowledge that the causality of these associations is not stringent. Some studies found that MEF_50_ was below average in subgroups of healthy never smokers [4, 5], or asymptomatic subjects [28]. Further, higher body mass index levels may attenuate MEF [29], but we observed no relation between body mass index levels and low MEF_50_ (Table 1).

### Study limitations

The extensive pulmonary function testing with spirometry, bodyplethysmography, and CO diffusion measurements was performed only once at baseline, thus, time-dependent effects could not be addressed. Because histological investigations or chest CT scans were not available in the GP setting of the current study, we were unable to assess small airways disease on a more pathophysiological basis. Further restrictive disorders might also account for MEF reduction. Patients with pulmonary restriction were not excluded in this study however, the prevalence in total cohort was low (3.1 %, N = 5) and there was no between group difference between patients with and without a MEF50 < 60 %.

## Conclusions

In older community-dwelling patients with pulmonary symptoms but without central airway obstruction a low maximum MEF, easily obtained by spirometry, showed clinical utility to identify patients with previously undiagnosed heart failure and patients at risk for pulmonary events. Thus, MEF_50_ values should also be evaluated when obtaining spirometry in persons aged >65 years with shortness of breath.

## Abbreviations

CI: Confidence intervals
COPD: Chronic Obstructive Pulmonary Disease
DLCO: Diffusion capacity of carbon monoxide
FEV1: Forced expiratory volume in 1 second
FVC: Forced vital capacity
GOLD: Global Initiative for Chronic Obstructive Lung Disease
GP: General practitioner
HF: Heart failure
HR: Hazard ratios
LVEF: Left ventricular ejection fraction
MEF_50_: Maximal expiratory flow at 50 % of the forced vital capacity
NT-proBNP: N-terminal pro-hormone B-type natriuretic peptide
PEF: Peak expiratory flow
PFT: Pulmonary function testing
RV: Residual volume
Rtot: Total resistance
SD: Standard deviation
TLC: Total lung capacity
